# The Differential Diagnosis between Brain Abscess and Sinus Thrombosis and the Treatment of These Conditions

**Published:** 1913-03

**Authors:** 


The Differential Diagnosis Between Brain Abscess and Sinus Thrombosis and the Treatment of These Conditions. Edward Bradford Dench (*Jour. Oph. and Oto-Laryng,* Dec., 1912), calls attention to two of the sequelæ of a suppurative process within the middle ear, brain abscess and thrombosis of the lateral sinus. Statistics compiled a number of years ago of ten thousand cases of middle ear suppuration, one patient in every 88 suffered from some intracranial complication. The importance of bearing in mind this possibility is evident. He takes up these two conditions separately and then emphasizes their points of differentiation. In brain abscess there are usually three distinct stages: (1) the stage of primary involvement; (2) the latent stage; (3) the terminal stage. When the brain substance becomes infected there is a sudden and abrupt rise in temperature, an increase in the pulse rate, and a certain amount of malaise. The temperature ranges from 101 to 103, seldom higher, and the pulse rate is proportionate. The rapid pulse is present only in the early stage. Even in acute abscess it is soon succeeded by a slow pulse, and this is one of the important signs to differentiate this condition from some of the other intracranial complications After from two to ten days the latent stage ensues, and this may last from a few weeks to a number of years Then follows the terminal stage in which the symptoms may be either local or general. Going more into detail there are certain signs which are indicative of intracranial inflammation no matter where located. These are headache, vomiting, muscular paralyses, and choked disc. Headache is always present in brain abscess. A persistent headache in a case of acute inflammation of the middle ear always suggests early intracranial involvement. Vomiting is present in about 50 per cent. of the cases. The muscular paralyses are usually those involving the ocular muscles, and the sixth nerve is the one most commonly affected.

Optic neuritis is a sign of considerable importance and in every suspected case the fundi should be examined.

He then describes certain localizing symptoms such as the aphasia present in involvement of the left temporosphenoidal lobe, and mirror writing, and, in cases of cerebellar involvement, disturbances of equilibrium.

He then discusses sinus thrombosis, giving an outline of the anatomy of the lateral sinus with some of its anomalies. The most characteristic symptom is a sudden elevation of temperature to 104 or 105 or even 106. This rise attains its maximum in from 2 to 6 hours and then suddenly falls either to normal or to 100 to 101. Soon a second similar rise occurs indicating a second amount of poison thrown into the general circulation. These rises and remissions take place once or twice in the twenty-four hours. Occasionally the fever remains persistently high. The characteristic temperature alone is sufficient to enable us to make a diagnosis in the very large majority of cases. Other symptoms are characterized by their absence. These patients do not seem to be ill. The sensorium is almost always clear, and there is practically no discomfort. If the case is untreated and the thrombus extends to the internal jugular vein other symptoms appear. There may be pain in the neck, and usually, torticollis. A very important sign is enlargement of the glands along the border of the sternomastoid. It is present in the early stages and should not be overlooked. Later septic emboli may lodge in some of the viscera and give rise to special symptoms. Choked disc is not infrequent. The value of the differential blood count is problematical. A positive blood culture is of the great importance and practically clinches the diagnosis, but the absence of a positive culture does not exclude the presence of this condition. Clinical signs should always outweigh a negative blood culture.

The differential diagnosis between these two conditions ordinarily should be easy. The characteristic difference in temperature, the absence of cerebral symptoms in sinus thrombosis and their presence in abscess, the rapid pulse in sinus thrombosis and the slow rate in every stage of abscess except the early, usually make it simple. Certain cases occur in which the abscess is secondary to a sinus thrombosis. These are confusing.

As regards the treatment, operation is the only plan that offers the patient the least hope of recovery.

He discusses his operative procedures in detail. It is better to err on the side of radicalism and save the patient than to be too conservative and thereby sacrifice a life.

